# Effects of Nano-SiO_2_ and Nano-CaCO_3_ on Mechanical Properties and Microstructure of Cement-Based Soil Stabilizer

**DOI:** 10.3390/nano15110785

**Published:** 2025-05-23

**Authors:** Baofeng Lei, Xingchen Zhang, Henghui Fan, Jianen Gao, Yichun Du, Yafei Ji, Zhe Gao

**Affiliations:** 1 Key Laboratory of Degraded and Unused Land Consolidation Engineering, the Ministry of Natural Resources, Xi’an 710075, China; lbf23@nwafu.edu.cn (B.L.); yichundu@yeah.net (Y.D.); yafeiji@yeah.net (Y.J.); 2 College of Water Resources and Architectural Engineering, Northwest A&F University, Yangling 712100, China; yt07@nwsuaf.edu.cn; 3 Northwest Engineering Corporation Limited, Power China, Xi’an 710065, China; 4 Institute of Soil and Water Conservation, Northwest A&F University, Yangling 712100, China; gjianen@yeah.net; 5 Yangling Vocational and Technical College, Yangling 712100, China; gaozhe2025@yeah.net

**Keywords:** soil stabilizer, nano-modification, compressive strength, flexural strength, stabilization mechanism

## Abstract

Soil stabilizers are environmentally friendly engineering materials that enable efficient utilization of local soil-water resources. The application of nano-modified stabilizers to reinforce loess can effectively enhance the microscopic interfacial structure and improve the macroscopic mechanical properties of soil. This study employed nano-SiO_2_ and nano-CaCO_3_ to modify cement-based soil stabilizers, investigating the enhancement mechanisms of nanomaterials on stabilizer performance through compressive and flexural strength tests combined with microscopic analyses, including SEM, XRD, and FT-IR. The key findings are as follows: (1) Comparative analysis of mortar specimen strength under identical conditions revealed that nano-SiO_2_ generally demonstrated superior mechanical enhancement compared to nano-CaCO_3_ across various curing ages (1–3% dosage). At 1% dosage, the compressive strength of both modified stabilizers increased with curing duration. Early-stage strength differences (3 days) remained below 3% but showed a significant divergence with prolonged curing: nano-SiO_2_ groups exhibited 10.3%, 11.3%, and 7.2% higher compressive strengths than nano-CaCO_3_ at 7, 14, and 28 days, respectively. (2) The strength enhancement effect of nano-SiO_2_ on MBER soil stabilizer followed a parabolic trend within 1–3% dosage range, peaking at 2.5% with over 15% strength improvement. (3) The exceptional performance of nano-SiO_2_ originates from its high reactivity and ultrafine particle characteristics, which induce nano-catalytic hydration effects and demonstrate strong pozzolanic activity. These properties accelerate hydration processes while promoting the formation of interlocking C-S-H gels and hexagonal prismatic AFt crystals, ultimately creating a robust three-dimensional network that optimizes interfacial structure and significantly enhances strength characteristics across curing periods. These findings provide scientific support for the performance optimization of soil stabilizers and their sustainable applications in eco-construction practices.

## 1. Introduction

As an eco-friendly engineering material capable of transforming loose soil into stabilized structures through chemical reactions, soil stabilizers are primarily categorized into inorganic, organic, ionic, and bio-enzymatic systems [1,2,3]. Among these, inorganic cement-based stabilizers have demonstrated superior technical maturity. They enhance soil mechanical strength and engineering stability by generating cementitious products through hydration reactions with soil mineral components [4,5]. Compared to other stabilizer types, this material exhibits distinct advantages, including construction convenience, strong environmental adaptability, and excellent consolidation stability [6,7]. Furthermore, owing to its abundant raw material availability and controllable costs, it has been widely implemented in road engineering, hydraulic facilities, and civil construction projects [8,9]. Particularly in resource-scarce regions, such as the Loess Plateau, such stabilizers hold special significance in balancing infrastructure demands with ecological preservation, delivering notable economic and environmental benefits [10].

With the escalating performance requirements in construction materials, recent research has focused on strength enhancement strategies for soil stabilizers [11,12,13]. Wang et al. [14] demonstrated through comparative experiments that loess treated with organic curing agents exhibits superior performance in both 7-day unconfined compressive strength and frost resistance compared to cement-based solidified soil. Specifically, the cement-based solidified soil showed a strength loss rate of 32.78% after 25 freeze-thaw cycles, while the organically treated soil demonstrated a significantly lower strength loss rate of 15.7%, indicating better frost resistance. Addressing regional soil characteristics, Yang et al. [15] revealed enhanced short-term mechanical responses of organic-stabilized clay in Northeast China under freeze-thaw cycles and variable compaction densities, outperforming conventional lime-based materials. In addition, Zhang et al. [16] conducted a pioneering investigation into the mechanistic effects of chloride salt environments on the performance of MBER-stabilized soils, revealing that an appropriate concentration of NaCl solution significantly enhances the early-stage strength of stabilized soils. Their findings demonstrate the applicability of NaCl solutions with concentrations below 1% in stabilized soil engineering construction, providing crucial theoretical support for the reinforcement technology of coastal saline soils. Wu et al. [17] systematically evaluated the water stability and mechanical properties of the metakaolin-cement composite system. Through parametric experiments, they determined the optimal blending ratio of the material, revealing that the threshold MK/cement ratio ranges from 1/3 to 1/2, which is attributed to the MK/hydrated CH ratio. Notably, Huang et al. [18] documented a 25% strength enhancement and 30% improvement in erosion resistance for alkali-activated slag stabilizers compared to conventional cement-treated soils, attributed to microstructural refinement.

Since American scientist Richard Feynman initiated research at the nanoscale in the 1960s [19], the application of nanomaterials in engineering has experienced rapid development. Compared with conventional materials, nanomaterials demonstrate remarkable advantages, exhibiting superior performance in strength, hardness, toughness, and plasticity [20], which has led to their gradual adoption in geotechnical engineering [21]. Kulkarni et al. [22] conducted experimental investigations on silty sand treated with colloidal nano-SiO_2_ (NS-20, NS-30, and NS-40) at three distinct concentration grades. The results demonstrated that the increase in cement content with different grades of nano-SiO_2_-treated soil played an indispensable role in improving the mechanical properties of soil. Optimal results were obtained for soil treated with NS-40 and 6% cement. Soaked CBR values of soil mixes were substantially enhanced by 240.76%, 268.62%, and 312.90%. Haeri et al. [23] investigated the reinforcement effects of nano-SiO_2_, nanoclay, and nano-CaCO_3_ on collapsible loess, revealing maximum strength improvements at 0.1% nano-SiO_2_, 0.2% nanoclay, and 0.4% nano-CaCO_3_ contents.

The aforementioned studies have systematically explored performance optimization pathways for traditional stabilization systems, establishing crucial theoretical foundations for engineering applications. Nevertheless, previous investigations predominantly focused on soil consolidation through nano-modified cement/concrete composites or direct utilization of nanomaterials as primary stabilizing agents to enhance soil mechanical properties. However, regarding novel nano-modified stabilizers, the mechanical characteristics and interfacial architectures of stabilized soils still require a comprehensive systematic investigation. Given the superior physicochemical properties of nanomaterials, these advanced additives demonstrate breakthrough potential in constructing high-performance and environmentally friendly stabilization systems. This study focuses on the synergistic enhancement mechanisms of nanomaterial-cement-based soil stabilizers. Through mortar strength comparative experiments, optimal nano-enhancing materials were screened. Combined with SEM, XRD, and FT-IR analyses, we elucidate the regulatory mechanisms of nanomodifiers on strength evolution in soil stabilizers. Subsequently, a novel nano-composite stabilization system integrating regional adaptability and low-carbon characteristics was developed, providing material innovation solutions for green geotechnical engineering construction.

## 2. Materials and Methods

### 2.1. Soil Stabilizer

The soil stabilizer employed in this study is MBER (Material Becoming Earth into Rock), an environmentally friendly cementitious composite developed by Gao Jian’en [24]. This inorganic binder system primarily consists of cement clinker, slag, gypsum, and core additives. The cement clinker in raw materials was procured from Qinlong Cement Plant in Xi’an, China; the fly ash consisted of Grade I power plant fly ash supplied by Zhengzhou Rongfeng New Materials Co., Ltd. (Zhengzhou, China); and the gypsum was laboratory-grade raw gypsum obtained from Guangdong Guanghua Technology Co., Ltd. (Shantou, China). The contents of ingredients in MBER and the SO_3_ content in cement clinker are shown in Table 1.

### 2.2. Nano-Modifiers

**Figure 1:**
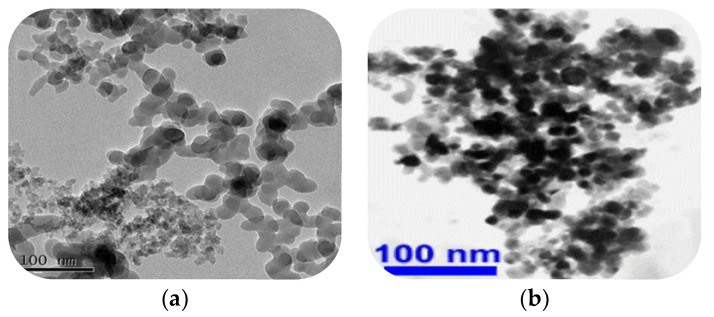
SEM image of nano-SiO_2_ and nano-CaCO_3_. (**a**) SEM image of nano-SiO_2_. (**b**) SEM image of nano-CaCO_3_.

**Figure 2:**
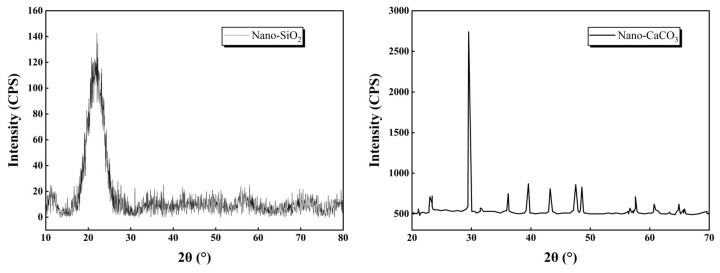
XRD image of nano-SiO_2_ and nano-CaCO_3_.

Through systematic evaluation of multifactorial influences, this investigation conducted a preliminary screening of nano-SiO_2_ and nano-CaCO_3_ as additives, selected based on their high reactivity, structural stability, cost-effectiveness, and broad applicability. The comparative analysis of strength enhancement effects in stabilized soil systems revealed distinct performance characteristics between these nanomaterials. The optimized formulation was ultimately determined through strength optimization trials, establishing an empirical basis for selecting nano-modified cementitious composites with superior mechanical performance. The nano-modifiers were procured from Shaanxi Dingyi Biotechnology Co., Ltd. (Xi’an, Chian), with corresponding microstructure micrographs in Figure 1, XRD patterns in Figure 2, and fundamental properties in Table 2. Both nano-SiO_2_ and nano-CaCO_3_ exhibited purities exceeding 99%, qualifying as laboratory-grade high-purity/high-reactivity nanomaterials.

### 2.3. Sample Preparation

**Figure 3:**
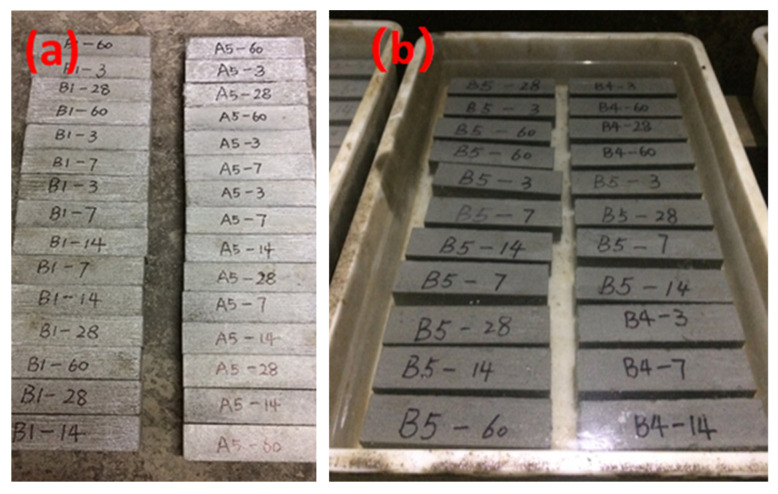
The Specimen after demolding (**a**) and the specimen under curing (**b**).

The nano-modifier contents were set at 1%, 1.5%, 2%, 2.5%, and 3% of the MBER total weight with reference to the literature [13], with untreated MBER serving as the control group. The MBER modified with nano-SiO_2_ and nano-CaCO_3_ nanoparticles were designated as the NS group and the NC group, respectively. The experimental procedure commenced with the addition of tap water to a mixer at a water-to-binder ratio of 0.5, followed by dispersion and homogenization of hydrophilic nanomaterials in the aqueous phase. Subsequently, pre-measured quantities of MBER stabilizer and ISO standard sand were incorporated into the mixture. The nano-SiO_2_ modified MBER mortar was prepared using a J-55 planetary mortar mixer (Xinmao Road Industry Testing Instruments Co., Ltd., Cangzhou, Hebei, China), followed by specimen molding with a ZS-15 cement mortar vibrator (Jinrui Testing Instruments Co., Ltd., Hengshui, Hebei, China). Figure 3a displays the demolded specimens. Triplicate specimens from each mold batch were distinctly labeled with different curing durations to minimize experimental variance. Following this protocol, the marked specimens underwent controlled hydration in a constant-temperature water tank maintained at 20 °C, with a 5 mm water layer maintained above the specimens as depicted in Figure 3b.

### 2.4. Test Procedures

To evaluate the mechanical performance of specimens, samples at each curing stage were initially subjected to flexural strength testing using a TYE-6A cement mortar flexural tester (Jingwei Instrument & Equipment Manufacturing Co., Ltd., Cangzhou, Hebei, China) with a controlled loading rate of 50 ± 10 N/s. Following fracture initiation, the resultant fragments were immediately transferred to a TYE-300 compression testing machine (Lukjian Testing Instruments Co., Ltd., Shijiazhuang, Hebei, China) for compressive strength evaluation, employing a displacement-controlled loading protocol at 2400 ± 200 N/s until structural failure. Quality assurance measures included six replicate flexural tests and six parallel compressive determinations per formulation, with the arithmetic mean of valid measurements adopted for statistical analysis to mitigate experimental variability.

**Figure 4:**
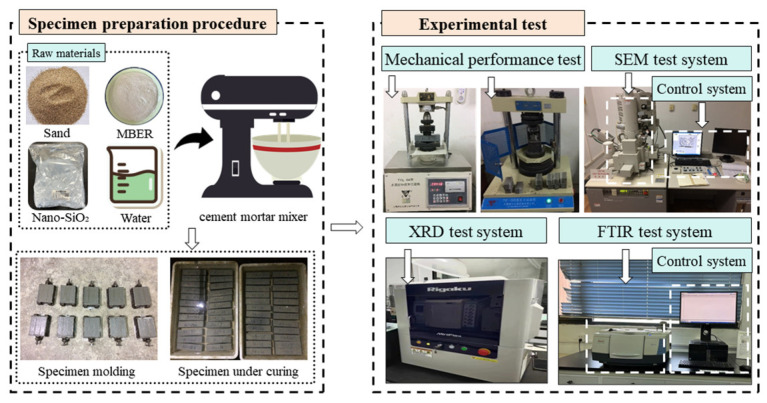
Sample preparation procedure and test methods.

Comparative microstructural characterization was conducted on specimens containing 12% nano-stabilizer versus MBER-stabilized controls to elucidate the interfacial modification mechanisms of nano-stabilized loess. The specimens were subjected to fracture surface observation and EDS analysis using an S-4800 field emission scanning electron microscope (Hitachi, Ltd., Tokyo, Japan) after 7-day and 28-day curing periods to investigate microstructural evolution and chemical composition variations. FTIR measurements were conducted with a Nicolet™ iS50R FTIR spectrometer (Tongce Technology Co., Ltd., Xi’an, China) to analyze chemical structures of hydration products. Phase analysis and crystallinity determination were performed via Rigaku Benchtop XRD (Miniflex600) (Bruker, Ltd., Ettlingen, Germany) with a scanning range of 2θ = 10–80° and scanning rate of 5°/min. Under controlled laboratory conditions, all experimental procedures were implemented at the Yangling National Agricultural Hi-tech Industrial Demonstration Zone, Shaanxi Province, China (Figure 4).

## 3. Results and Discussions

### 3.1. Strength of Different Cement-Based Soil Stabilizers

**Figure 5:**
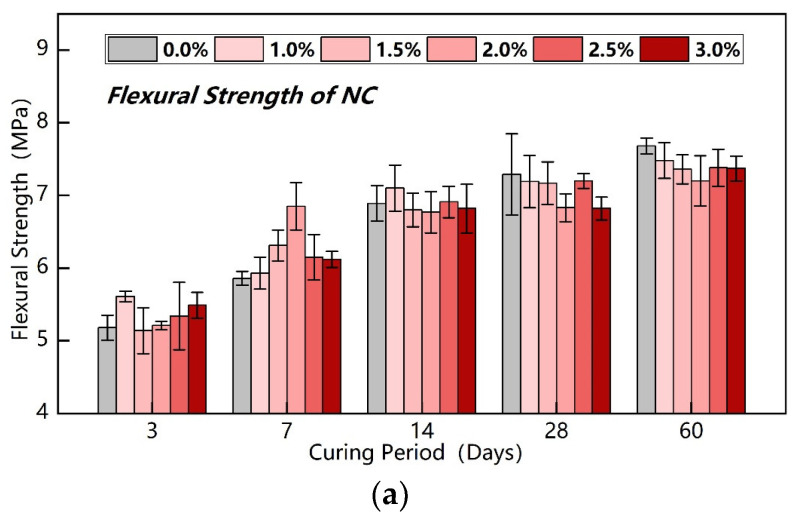

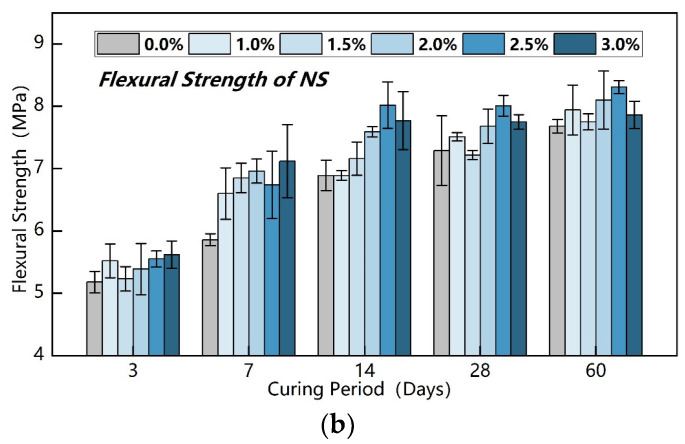
Flexural strength of NC and NS. (**a**) Flexural strength of NC. (**b**) Flexural strength of NS.

Figure 5 presents the flexural strength test results of nano-SiO_2_ and nano-CaCO_3_ under various dosages and curing ages. This study aims to elucidate the variation pattern of mortar strength with curing age for the same nanomaterial at different dosages by investigating the influence mechanism of nanomaterial dosage on the strength development of modified binder mortar, thereby evaluating the strength growth potential and application prospects of the material. As shown in Figure 5a, for the NC-modified binder groups, specimens with 1% NC dosage demonstrated significantly higher flexural strength than the control group at all curing ages except for marginally lower values at 28-day and 60-day ages. Specimens with 1.5% and 2% NC dosages exhibited slightly higher flexural strength than the control group during early and middle curing stages, but lower strength in later stages. Comparative analysis of different NC dosage groups reveals a general decreasing trend in later-stage mortar flexural strength as the dosage increases from 1% to 3%. These findings indicate that a 1% NC dosage significantly enhances the flexural strength of the binder, while dosages exceeding 2% demonstrate inhibitory effects on mortar flexural strength development, manifesting a negative growth trend. This suggests the existence of an optimal NC dosage around 1% for maximum flexural strength enhancement in cementitious materials. Excessive NC incorporation (>2%) adversely affects the flexural strength development of mortar, likely due to nanoparticle agglomeration and subsequent microstructural deterioration. Further analysis reveals that the strength enhancement mechanism at optimal dosage primarily derives from nano-filling effects and accelerated hydration reactions, whereas overdosage induces adverse interfacial effects between nanoparticles and cement matrix.

Figure 5 illustrates the flexural strength development of nano-SiO_2_ modified binder mortar under varying dosages. For NS-modified binder groups, specimens with 1–3% NS dosages exhibited significantly higher flexural strength compared to the 0% NS control group, demonstrating the substantial enhancement effect of NS incorporation on mortar flexural properties. A comparative analysis of NS dosage groups reveals a characteristic trend: the mortar flexural strength initially increases, then decreases with rising NS dosage from 1% to 3%, indicating the existence of an optimal NS dosage around 2% for maximum strength enhancement. Further examination of strength evolution shows a distinct performance pattern at 2.5% NS dosage. Notably, specimens with 2.5% NS dosage surpassed all other groups in flexural strength across all curing ages except at 7-day, suggesting this specific dosage achieves peak enhancement efficiency. This phenomenon implies that while 2% NS dosage generally demonstrates optimal performance within the conventional dosage range, the 2.5% dosage exhibits exceptional strengthening effects under prolonged curing conditions, likely attributed to improved nanoparticle dispersion and enhanced pozzolanic reaction efficiency at this critical concentration threshold.

**Figure 6:**
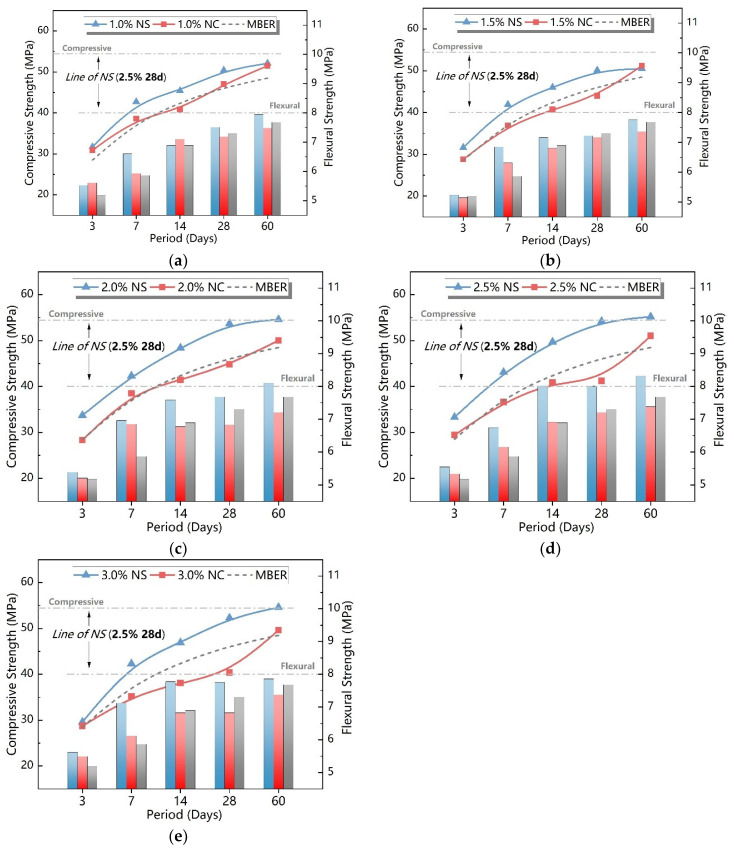
Comparison of compressive strength on NS and NC with different contents. (**a**) Nanomaterials with 1% content. (**b**) Nanomaterials with 1.5% content. (**c**) Nanomaterials with 2% content. (**d**) Nanomaterials with 2.5% content. (**e**) Nanomaterials with 3% content.

Figure 6 presents the comparative results of compressive and flexural strength in mortar specimens containing nano- SiO_2_ and nano- CaCO_3_ at various dosages. In order to highlight the enhancement effect of NS on the binder, the compressive and flexural strength values of specimens with 2.5% NS dosage were selected as reference benchmarks, as shown in Figure 6. Analysis revealed that both compressive and flexural strengths increased with extended curing ages, eventually stabilizing during later curing stages. For NS-modified specimens, strength values at all curing ages consistently surpassed those of the control group, demonstrating the effective improvement of the MBER soil binder through NS incorporation. In contrast, NC-modified specimens exhibited lower strength than the control group during initial curing stages, with strength superiority emerging only after prolonged aging. This behavior indicates that NC primarily contributes to mid-to-late stage strength development in cementitious systems.

Figure 6 demonstrates the comparative performance of nano-SiO_2_ and nano-CaCO_3_ modified cementitious binders under equivalent dosages. The NS-modified groups exhibited significantly superior enhancement effects compared to the NC-modified groups. Notably, the strength of NC-modified binders decreased below that of the control group with increasing NC dosage, indicating that excessive NC addition inhibits strength development. The strength enhancement effect of NC on cement-based binders showed significant fluctuations, with overdosage causing strength suppression. Mechanistic analysis reveals three primary factors: First, during material preparation, the hydrophobic nature of NC resulted in poor dispersion performance and inferior homogeneity to NS in mortar specimens. Second, at the microstructural level, although NC particles possess a specific surface area 200–400 times larger than binder particles, this remains orders of magnitude lower than NS, leading to weaker surface energy, reduced intermolecular interactions, and limited van der Waals agglomeration effects between particles. Lastly, regarding hydration reactions, while NC’s ultrafine particle size and high fluidity enable pore-filling and provide nucleation sites for early hydration, its lack of high pozzolanic activity results in significantly weaker enhancement of binder hydration compared to NS.

### 3.2. Correlation Analysis of Compressive and Flexural Strengths

**Figure 7:**
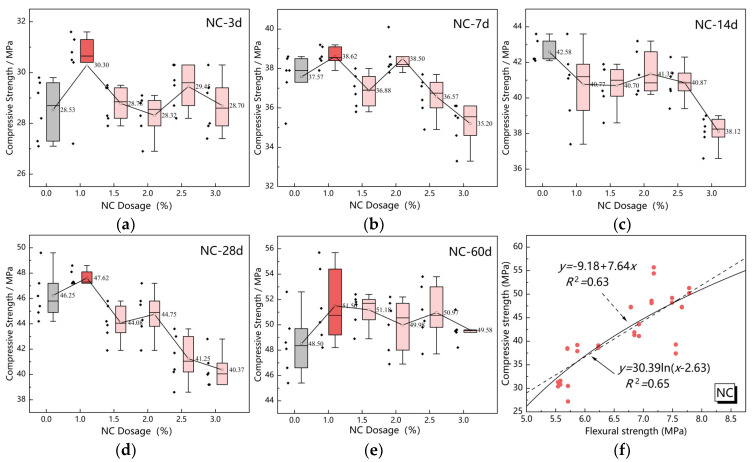
Correlation analysis of compressive and flexural strength of NC. (**a**) NC-3d. (**b**) NC-7d. (**c**) NC-14d. (**d**) NC-28d. (**e**) NC-60d. (**f**) Correlation analysis of NC.

**Figure 8:**
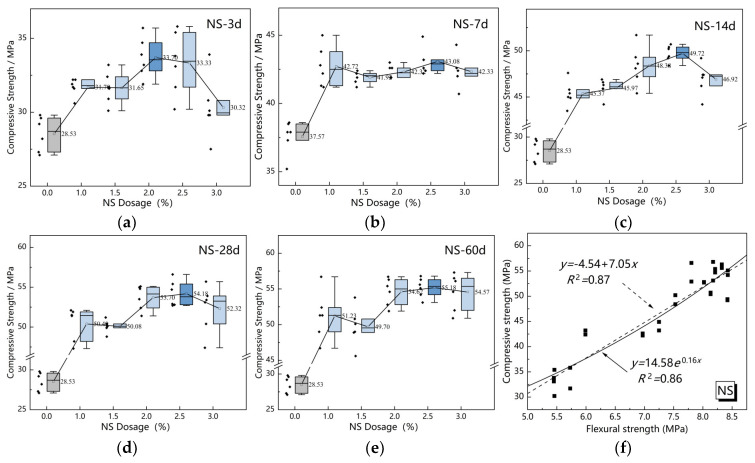
Correlation analysis of compressive and flexural strength of NS. (**a**) NS-3d. (**b**) NS-7d. (**c**) NS-14d. (**d**) NS-28d. (**e**) NS-60d. (**f**) Correlation analysis of NS.

Figure 7 and Figure 8 present fitting analyses of the correlation between compressive and flexural strength for the two nanomaterials, based on mean values of experimental results.

The analysis of Figure 7 reveals that in NC-modified specimens, the inherent hydrophobicity and poor dispersibility of nano-calcium carbonate induce agglomeration within mortar specimens, resulting in microstructural heterogeneity. This structural non-uniformity leads to a weaker linear correlation between flexural and compressive strength (*R*^2^ = 0.63), necessitating supplementary explanation of data variance through a logarithmic model (*R*^2^ = 0.65).

Figure 8 analysis reveals that in NS-modified specimens, the high pozzolanic activity and pore-filling effects of nano-silica significantly enhance material compactness. The homogeneous dispersion characteristics establish a strong linear correlation between flexural and compressive strength (*R*^2^ = 0.87), while the exponential model (*R*^2^ = 0.86) further confirms NS’s pronounced acceleration of hydration reactions. Furthermore, NS’s specific surface area substantially exceeds that of NC, accompanied by enhanced surface energy and van der Waals forces, which promote the formation of more stable microstructural configurations. This mechanistic understanding explains the concentrated data distribution and superior goodness-of-fit observed in NS specimens.

A comprehensive analysis of both modifiers’ strength correlations demonstrates that NS-modified MBER soil binder is particularly suitable for applications requiring high strength synergy, such as transportation concrete pavements. The high goodness-of-fit (*R*^2^ > 0.85) indicates reliable prediction of compressive strength through flexural strength measurements. Conversely, NC-modified systems exhibit greater strength variability due to their physical interaction dominance and dispersion deficiencies. Therefore, practical applications should prioritize modifier selection based on performance requirements, coupled with dosage optimization to achieve balanced mechanical properties.

### 3.3. Microscopic Test Results and Analysis

#### 3.3.1. XRD Pattern Analysis

**Figure 9:**
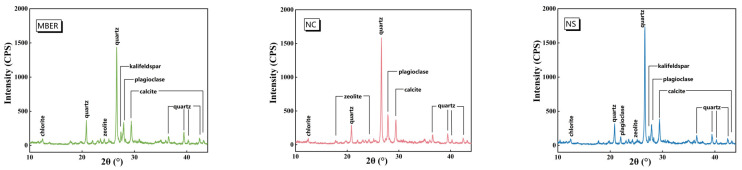
XRD of stabilized soil.

Microcomposition analysis was conducted on cross-sections of MBER-, NC-, and NS-stabilized soil specimens after 28-day curing to investigate the effects of nanomaterials on the mineral composition and content within stabilized soils. XRD analysis using an infrared diffractometer revealed the crystalline phases, with primary mineral types and contents determined through Bragg angle peak position analysis, as shown in Figure 9. The three stabilized soils exhibited identical clay mineral compositions dominated by illite and chlorite, alongside non-clay minerals including quartz, calcite, and feldspar. A comparative peak analysis demonstrated that NS-stabilized soil achieved the highest quartz content, while sharing comparable calcite, illite, and chlorite contents with NC-stabilized soil but containing less feldspar. MBER-stabilized soil displayed minimal quartz content with non-clay/clay mineral proportions analogous to NC-stabilized soil. XRD patterns indicate that nano-SiO_2_ incorporation induced negligible mineralogical alterations but modified inert mineral packing and cementation patterns through particle size modulation, structural unit reorganization, and phase interface optimization. These microstructural adjustments activated mineral surface interactions, enhanced hydration-hydrolysis reactions, and consequently altered macroscopic physicochemical properties [13].

#### 3.3.2. FT-IR Results and Analysis

**Figure 10:**
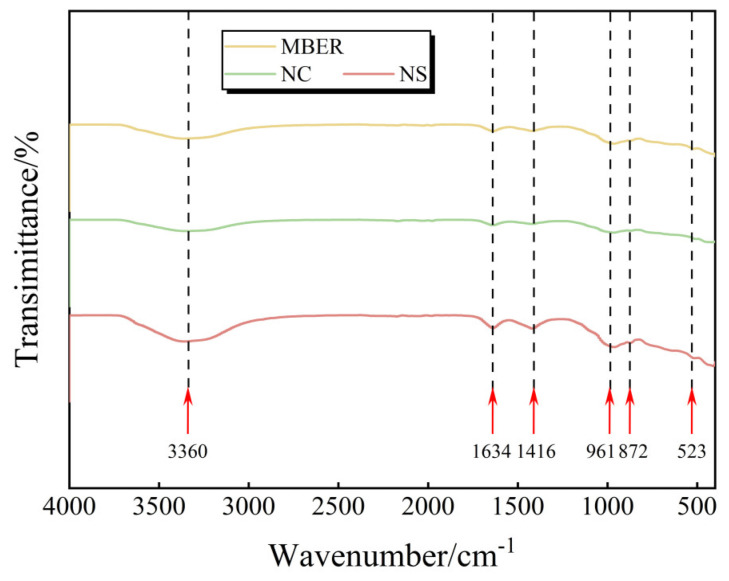
FT-IR pattern of the specimen.

Figure 10 presents the FT-IR spectra of MBER-, NC-, and NS-stabilized soils at 28-day curing. The analysis reveals a broad absorption band near 3360 cm⁻^1^ corresponding to O–H stretching vibrations in Si–OH groups, indicating surface-adsorbed water molecules. A minor peak observed at 1634 cm⁻^1^ is attributed to H–O–H bending vibrations of water molecules, suggesting the gradual conversion of free water into crystalline water through hydration reactions with extended curing [25,26]. The absorption band at 1416 cm⁻^1^ corresponds to asymmetric stretching and bending vibrations of C–O bonds in carbonates, potentially associated with CO_2_ absorption from ambient air during specimen preparation [27]. Characteristic SiO_2_ bands are identified at 961 cm⁻^1^ (asymmetric stretching vibration of Si–O–Si), 872 cm⁻^1^, and 523 cm⁻^1^ (bending vibrations of Si–O bonds). Additionally, due to the high quartz content in loess, the Si–O–Ca (Al) vibrational band of C-(A)-S-H near 1000 cm⁻^1^ is obscured within the dominant Si–O vibrational region [28].

#### 3.3.3. Microstructure Analysis

**Figure 11:**
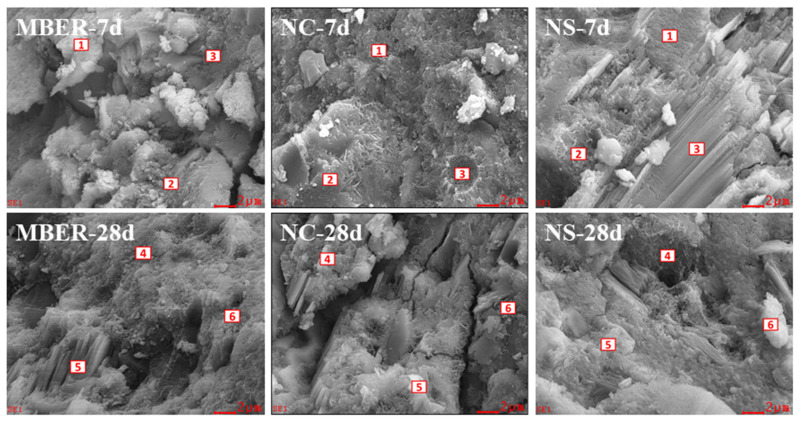
SEM and EDS of different stabilized soils.

**Figure 12:**
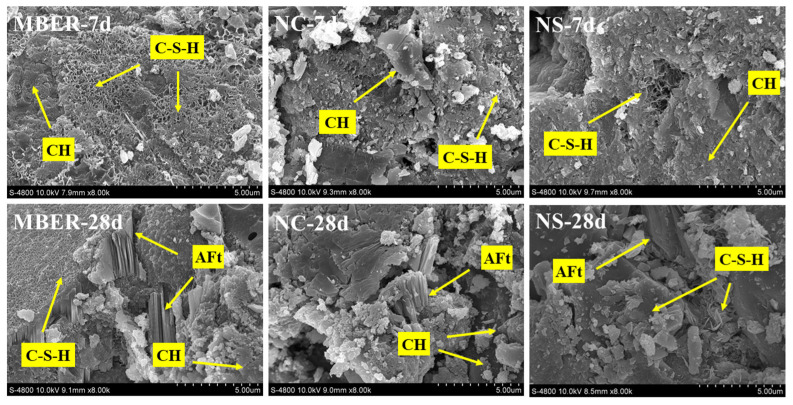
SEM of different stabilized soils.

The specimens were cured to designated ages, followed by interfacial microstructure observation and EDS analysis of fractured surfaces for MBER-stabilized soil, NC-stabilized soil, and NS-stabilized soil using an S-4800 field emission scanning electron microscope. By investigating phase interface structures and crystalline composition variations, this study examines the interfacial reconstruction process of nano-stabilized soil units and their influencing factors, explores the impact of nano-silica’s high pozzolanic activity on hydration processes, and analyzes the microscopic consolidation mechanisms. Analytical results are presented in Figure 11 and Figure 12 and Table 3.

Figure 11 and Figure 12 illustrate the microstructural evolution characteristics of three stabilization materials at different curing stages. Table 3 displays EDS results corresponding to Figure 9. At 7 days of curing, MBER-stabilized soil exhibited fibrous or acicular calcium silicate hydrate (C-S-H) gels alongside coarse laminar portlandite (CH) crystals. With extended curing duration, abundant three-dimensional C-S-H networks interwoven with hexagonal prismatic ettringite (AFt) were observed, accompanied by reduced CH content. The EDS analysis revealed increased O^2−^ content and decreased Si^4+^ content in MBER-stabilized colloids from 7 to 28 days, indicating enhanced oxygen-silicon ratio during hydration. This suggests the transformation of silica crystals into free silicate ions (SiO_3_^2−^), which accelerates the hydration reactions of tricalcium silicate and tricalcium aluminate, thereby promoting C-S-H formation and improving macroscopic performance. Concurrently, significant Ca^2+^ reduction implied the conversion of free calcium ions into aggregated C-S-H gels during hydration [29,30,31]. Notably, the SEM observations of NS specimens at 7-day curing identified pronounced nano-SiO_2_ agglomeration. This phenomenon primarily stems from the material’s intrinsic physicochemical properties—the exceptionally high specific surface area and surface energy of nano-SiO_2_ particles render them thermodynamically favorable to cluster, thereby reducing interfacial energy through particle aggregation [32]. Concurrently, the short curing duration imposes kinetic limitations: insufficient time for nanoparticle dispersion synergizes with evolving hydration kinetics. During early curing stages, progressive formation of cementitious hydration products creates physical confinement effects that restrict particle mobility and spatial reorganization, further exacerbating localized agglomeration [32].

A comparative analysis of NC- and NS-stabilized soils revealed that nano-silica incorporation effectively refined CH crystals during early hydration, generating continuous three-dimensional C-S-H networks. NC-stabilized soil primarily formed C-S-H gels in early stages with limited AFt formation and low hydration product density in later phases, suggesting possible hydration inhibition from high nano-calcium carbonate content. In contrast, NS-stabilized soil exhibited diversified C-S-H morphologies with minimal CH content at 7 days, progressing to complete CH consumption and forming spatial networks of radial/3D C-S-H intergrown with AFt by 28 days. Comprehensive analysis demonstrates that NS incorporation promotes synergistic growth of multi-phase hydration products through optimized interfacial contact modes. The resultant three-dimensional network architecture significantly enhances inter-particle bonding strength, providing microstructural explanations for the mechanical improvement mechanisms in NS-stabilized soils [33,34,35].

### 3.4. Discussions

The MBER soil stabilizer is a powdered material produced by blending and grinding cement clinker with core raw materials. This environmentally friendly inorganic cementitious material demonstrates capabilities for consolidating general soils at ambient temperature, exhibiting high consolidation strength, durability, minimal deformation, and broad applicability. Its pozzolanic reactions primarily involve tricalcium silicate (C_3_S), dicalcium silicate (C_2_S), tricalcium aluminate (C_3_A), and tetracalcium aluminoferrite (C_4_AF) [3,4,5,13,14,26]. These reactions produce hydration products including calcium silicate hydrate (C-S-H gel), ettringite (AFt), calcium hydroxide (CH), and carbonation products like calcium carbonate (CaCO_3_), accompanied by heat release, as illustrated in Equations (1)–(4) [36,37,38,39].

3Ca∙SiO_2_ + mH_2_O = xCaO∙SiO_2_∙yH_2_O + (3 − x) Ca(OH)_2_ (1)

2Ca∙SiO_2_ + mH_2_O = xCaO∙SiO_2_∙yH_2_O + (2 − x) Ca(OH)_2_ (2)

3CaO∙Al_2_O_3_ + 6H_2_O = 3CaO∙Al_2_O_3_∙6H_2_O (3)

4CaO∙Al_2_O_3_∙Fe_2_O_3_ + 7H_2_O = 3CaO∙Al_2_O_3_∙6H_2_O + CaO∙Fe_2_O_3_∙H_2_O (4)

As a novel nano-stabilization material, nano-modifiers exhibit hydration processes encompassing both conventional cementitious reactions and enhanced physicochemical complexity due to the incorporation of nano-silica particles with ultrafine particle size, high pozzolanic activity, and improved fluidity [40,41,42]. The nano-silica demonstrates nano-induced hydration effects and strong pozzolanic reactivity owing to its high surface activity and ultrafine characteristics. It participates in secondary hydration with early-stage C3S products, accelerates the hydration process, and rapidly reacts with CH through silica-core activation, generating abundant C-S-H gels with diverse morphologies. Notably, nano-SiO_2_ agglomeration emerges during incipient hydration stages, arising from the particles’ intrinsic high specific surface area and elevated surface energy that promote spontaneous clustering through thermodynamic surface energy minimization mechanisms [32]. The observed variability in strength enhancement of nano-CaCO_3_ in cement-based stabilizers, where excessive nano-CaCO_3_ even inhibits strength development, primarily stems from three interrelated factors. First, the hydrophobic nature of nano-CaCO_3_ leads to inferior dispersion uniformity compared to nano-SiO_2_ during mortar specimen preparation. Second, despite nano-CaCO_3_ exhibiting 200–400 times higher specific surface area than stabilizer particles, its surface area remains orders of magnitude lower than nano-SiO_2_, resulting in reduced surface energy, weaker intermolecular interactions, and less effective particle aggregation through van der Waals forces. Third, although nano-CaCO_3_ enhances void-filling efficiency and accelerates early hydration via nucleation effects due to its ultrafine particle size and high fluidity, its limited pozzolanic activity significantly diminishes catalytic effectiveness in hydration processes compared to nano-SiO_2_ [28,43,44]. SEM analysis further demonstrates that NS incorporation effectively refines CH crystals (detrimental to strength development) and promotes multi-morphology C-S-H gel formation within hardened matrices. These gels interlock with hexagonal prismatic AFt crystals to establish robust three-dimensional cementitious networks, thereby optimizing the interfacial architecture of nano-modified stabilizers and significantly enhancing their age-dependent mechanical performance [40,41].

Integrated analysis reveals that incorporating appropriate nano-SiO_2_ dosage enriches hydration products within stabilized soil colloids and optimizes phase interface structures to facilitate spatial network formation. This fundamental alteration in phase contact characteristics between soil units significantly enhances the microstructural configuration, thereby improving the strength of stabilized soil specimens.

## 4. Conclusions

This study investigates MBER soil stabilizer modified with nano-CaCO_3_ and nano-SiO_2_ additives. Through comparative analysis of their strength enhancement effects, an optimized modification strategy is proposed. The main conclusions are as follows:

(1) Mortar specimen tests under identical conditions demonstrate that NS-modified stabilizers with 1–3% dosage generally outperform NC-modified counterparts across all curing ages. This confirms NS as the superior nanomaterial for enhancing MBER-based stabilizers within this dosage range.
(2) The strength enhancement of NS-modified stabilizers exhibits a parabolic trend within 1–3% dosage, peaking at 2.5% addition with strength enhancement exceeding 15%. This optimal dosage effectively improves the mechanical performance of MBER stabilizers.
(3) The high reactivity and ultrafine particle characteristics of NS induce nano-activated hydration effects and strong pozzolanic activity, accelerating the hydration process. NS effectively refines calcium hydroxide (CH) crystals detrimental to strength development while promoting multi-morphology calcium silicate hydrate (C-S-H) gel formation. These gels interlock with hexagonal prismatic ettringite (AFt) crystals to establish robust three-dimensional cementitious networks, thereby optimizing interfacial microstructure and enhancing age-dependent mechanical properties.

## Figures and Tables

**Table 1:** Soil stabilizer ingredients and SO_3_ content in the test.

Ingredients	Cement Clinker	Fly Ash	Gypsum	Active Agent	Soil Stabilizer
Content %	85	11	3	1	100
SO_3_ content	2.01	0.26	1.40	0	3.67

**Table 2:** Basic physical properties of nano-SiO_2_ and nano-CaCO_3_.

Physical Property	Purity (%)	APS (nm)	SSA (m^2^/g)	Bulk Density (g/cm^3^)	True Density (g/cm^3^)
Nano-SiO_2_	99.9%	30	600	0.08	2.2~2.6
Nano-CaCO_3_	99.9%	20	50	0.30	5.7~5.8

**Table 3:** Energy spectrum analysis results of different stabilized soils.

Sample	Point	Metering Mode	C	O	Na	Mg	Al	Si	S	K	Ca	Fe	Mineral Type
MBER	1	Wt%	4.58	41.33	0.75	0.78	2.75	18.75	0.68	1.26	27.32	1.81	CH
	2	At%	8.35	56.58	0.71	0.7	2.23	14.62	0.46	0.71	14.93	0.71	CH
	3	Wt%	6.3	47.11	1.02	0.58	2.67	12.72	0.52	1.25	26.47	1.36	C-S-H
	4	At%	10.88	41.06	0.92	0.49	2.05	29.4	0.34	0.66	13.69	0.51	C-S-H
	5	Wt%	5.65	41.75	0.39	0.4	2.13	28.23	0.33	0.76	19.39	0.97	AFt
	6	At%	9.95	55.19	0.36	0.34	1.67	21.26	0.22	0.41	10.23	0.37	C-S-H
NC	1	Wt%	4.35	41.43	1.15	1.00	4.17	10.95	1.09	1.05	32.12	2.69	C-S-H
	2	At%	8.05	57.58	1.11	0.92	3.43	8.67	0.76	0.6	17.82	1.07	CH
	3	Wt%	4.33	41.48	0.98	1.11	4.20	11.22	1.14	0.96	32.15	2.43	C-S-H
	4	At%	8.01	57.58	0.94	1.02	3.46	8.87	0.79	0.55	17.82	0.97	C-S-H
	5	Wt%	4.17	41.30	1.07	0.86	4.27	11.09	1.13	1.17	32.46	2.49	AFt
	6	At%	7.74	57.58	1.04	0.79	3.53	8.81	0.78	0.67	18.07	0.99	CH
NS	1	Wt%	4.74	38.78	1.13	0.7	3.46	13.32	0.78	1.64	33.4	2.05	CH
	2	At%	8.9	54.65	1.11	0.65	2.89	10.7	0.55	0.95	18.79	0.83	C-S-H
	3	Wt%	4.75	38.93	1.11	0.62	3.38	13.27	0.68	1.58	33.54	2.15	AFt
	4	At%	8.91	54.85	1.09	0.57	2.82	10.65	0.48	0.91	18.86	0.87	C-S-H
	5	Wt%	4.34	37.99	0.99	0.63	3.55	13.11	0.88	1.71	33.36	1.99	AFt
	6	At%	8.29	54.51	0.99	0.6	3.02	10.72	0.63	1	19.11	0.82	C-S-H

## Data Availability

Data are contained within the article.
